# Oral Health Intervention Strategies for Self‐Caring Adults With Disabilities: A Systematic Review

**DOI:** 10.1155/ijod/8819298

**Published:** 2026-01-21

**Authors:** Nithimar Sermsuti-Anuwat, Palinee Hongpaitoon, Daophon Ardiam

**Affiliations:** ^1^ Faculty of Dentistry, Chulalongkorn University, Bangkok, Thailand, chula.ac.th; ^2^ Faculty of Dentistry, Western University, Pathum Thani, Thailand, western.ac.th

**Keywords:** adult, dental care for disabled, oral health, oral hygiene, public health, self care

## Abstract

**Background:**

Oral health disparities among adults with disabilities remain a significant challenge.

**Objective:**

To systematically evaluate the effectiveness of educational, behavioral, and pharmacological strategies intended to enhance oral health self‐care among independent adults with disabilities.

**Methods:**

This systematic review adhered to PRISMA 2020 recommendations and was registered with PROSPERO (CRD420251015528). We identified randomized controlled trials (RCTs) published in English from 2000 to 2024 through extensive searches of PubMed, Scopus, EBSCOhost, the Cochrane Library, ProQuest, and relevant gray literature sources. Studies were eligible if they involved adults aged 18–70 years with psychiatric, neurological, intellectual, or physical disabilities who were able to perform their own oral hygiene. Interventions included educational, behavioral, or pharmacological strategies compared to any control. Two reviewers independently completed the screening and data‐extraction processes and appraised study quality using the Cochrane RoB 2.0 tool. Narrative synthesis was conducted due to heterogeneity.

**Results:**

Ten RCTs involving 681 participants from diverse countries met the inclusion criteria. Educational and behavioral interventions, including oral hygiene instruction (OHI), visual aids, hands‐on demonstration, and motivational interviewing (MI), significantly improved oral health knowledge, attitudes, and self‐care practices in adults with disabilities. These interventions were particularly effective when tailored to the specific needs of participants and delivered with ongoing reinforcement. Pharmacological strategies, primarily chlorhexidine‐based adjuncts, further reduced plaque, gingival bleeding, and oral microbial load, especially when combined with education or behavioral support. While most interventions produced significant short‐term improvements in clinical and behavioral outcomes, overall certainty of evidence was limited by heterogeneity in study designs, intervention protocols, and risk of bias.

**Conclusions:**

Multifaceted interventions that combine tailored education, behavioral reinforcement, and pharmacological support improve oral health outcomes for independent adults with disabilities. Future research should prioritize standardized methodologies, rigorous risk‐of‐bias assessment, and long‐term follow‐up to strengthen the evidence base and guide clinical practice and policy.

## 1. Background

The World Health Organization (WHO) defines disability as “the umbrella term for impairments, activity limitations, and participation restrictions, referring to the negative aspects of the interaction between an individual (with a health condition) and that individual’s contextual factors (environmental and personal factors)” [1]. Globally, ~1.3 billion individuals, representing nearly 16% of the global population, are living with significant disability [2]. This population encompasses a broad spectrum of conditions, which can be subcategorized into physical (e.g., mobility impairments), intellectual/developmental, psychiatric, and sensory disabilities [1, 3].

A growing body of evidence demonstrates that adults with disabilities consistently face poorer overall health outcomes compared to the general population [4, 5]. This disparity extends to oral health, where individuals with disabilities experience a similar risk for oral diseases, yet often face greater severity and faster progression [6]. Recent epidemiological data quantify this burden; for instance, studies indicate that adults with intellectual and developmental disabilities exhibit significantly higher rates of untreated dental caries and periodontal disease compared to the general population, with the prevalence of periodontitis reported as high as 80% in some adult cohorts [7]. Furthermore, adults with disabilities are more likely to have missing teeth and fewer filled teeth, reflecting a pattern of extraction over restoration [8]. Multiple factors contribute to these outcomes, including inadequate oral hygiene, limited access to dental care, and the impact of coexisting medical conditions [9].

In the context of this review, we specifically focus on “independent adults with disabilities.” We define this group as individuals aged 18 and older who retain the physical and cognitive capacity to perform Activities of Daily Living (ADLs), specifically oral self‐care, with minimal or no physical assistance (e.g., scoring as independent on the Barthel Index or requiring only verbal prompting) [10]. Despite their independence, these adults often encounter unique barriers to maintaining oral health, such as impaired fine motor skills, sensory sensitivities, and inequitable access to healthcare systems [11]. Dental professionals also face challenges in delivering effective treatment and preventive care to this population [12].

Various oral health interventions are used to address these challenges. Educational strategies aim to improve knowledge and self‐care skills, behavioral interventions target psychological and social factors that influence oral hygiene habits, and some programs incorporate physical exercises or therapy to enhance function [13, 14]. Pharmacological approaches, such as the use of chemical or antimicrobial agents like chlorhexidine, may also be employed to support oral health outcomes [15].

However, a gap analysis of the current literature reveals that adult‐specific research is scarce. The majority of existing studies and systematic reviews have focused primarily on children [16, 17], adolescents with disabilities [18, 19], or dependent older adults [20, 21]. For example, a recent review of oral care interventions for autistic individuals included only one study involving autistic adults [22]. Many studies group together mixed‐aged individuals with disabilities, which further limits the applicability of findings to adult populations [22–24]. Consequently, independent adults with disabilities remain an underrepresented and underserved group in oral health research [25–28]. This oversight is especially significant given their distinct challenges in maintaining oral hygiene and achieving optimal oral health [6, 9, 11].

Addressing oral health in this population has broader implications. Effective oral health interventions can improve not only physical well‐being but also foster self‐esteem, enhance social participation, and reduce caregiver burden [11]. Improving the oral health of independent adults with disabilities may also contribute to greater health equity and inform public health strategies targeting vulnerable groups.

Therefore, the aim of this systematic review is to evaluate existing evidence on effective oral health interventions specifically targeted at independent adults with disabilities who are capable of performing their own oral care. By addressing this gap, this review seeks to inform the formulation of tailored, evidence‐based strategies to enhance oral health outcomes and overall well‐being for this vulnerable and often overlooked population.

## 2. Materials and Methods

### 2.1. Research Design

This systematic review followed the PRISMA 2020 guidelines [29], and the protocol was registered in PROSPERO (CRD420251015528), available at https://www.crd.york.ac.uk/PROSPERO/view/CRD420251015528.

### 2.2. Identifying the Question

This review addressed the following research question: What effective oral health interventions have been implemented for independent adults with disabilities capable of performing their own oral hygiene?

### 2.3. Eligibility Criteria

The search strategy and inclusion criteria were developed using the PICOS framework, which outlines Population, Intervention, Comparator, Outcome, and Study Design components [30].

• Participants: Adults with disabilities or special care needs (e.g., psychiatric disorders, neurological conditions, intellectual/developmental disabilities, and physical disabilities), aged 18–70 years or with a mean age within this range. Participants had to be capable of self‐care independently or with minimal caregiver assistance. Studies were included if at least 75% of participants met the specified age criteria.
• Intervention: Eligible interventions aimed at improving oral health self‐care and hygiene practices, including:
  • Educational/behavioral interventions: Programs focused on oral health education (OHE), disease prevention, and behavioral change strategies addressing psychological and social factors. These may also include physical exercises aimed at improving oral functional performance, such as facial muscle training, tongue exercises, and salivary gland massage [13, 14].
  • Pharmacological intervention/antimicrobial therapy: Use of chemical or antimicrobial agents such as chlorhexidine in oral hygiene regimens [15].
• Comparator: Any comparator group was acceptable, including those receiving no intervention, standard care, or alternative interventions.
• Outcome: Studies needed to report at least one of the following:
  • Clinical outcomes (e.g., oral hygiene status, plaque levels, and gingival health),
  • Behavioral outcomes (e.g., tooth brushing frequency and adherence),
  • Knowledge and attitudinal outcomes (e.g., improvement in oral health knowledge or attitudes).
• Study design: Eligibility was restricted to randomized controlled trials (RCTs).

#### 2.3.1. Inclusion Criteria

Eligible studies were full‐text, peer‐reviewed publications in English from 2000 to 2024.

#### 2.3.2. Exclusion Criteria

Studies were excluded when the study population did not align with the defined eligibility criteria (e.g., children, dependent individuals, or those without disabilities) or if no oral health self‐care intervention or relevant outcome was reported. Additionally, non‐RCTs, quasiexperimental studies, pilot/feasibility studies, expert opinions, reviews, qualitative studies, and non‐English articles were excluded.

### 2.4. Information Sources

Search was conducted by three reviewers (NS, PH, and DA) in September 2024 across PubMed, Scopus, EBSCOhost (CINAHL), the Cochrane Library, and ProQuest. Gray literature was explored through OpenGrey and Google Scholar. Manual reference checks were also performed.

### 2.5. Search Strategy

Search strategies combined MeSH terms, synonyms, and free‐text keywords relevant to “disability” and “oral health,” with filters for RCTs. Full search strings for each database are provided in Table 1.

**Table 1:** The search strategy employed in the review.

Databases	The search strategy
PubMed	((“disabilit ^∗^”[Title/Abstract] OR “impair ^∗^”[Title/Abstract] OR “Disabled”[Title/Abstract]) AND (“Oral health”[Title/Abstract] OR “Dental health”[Title/Abstract] OR “Oral hygiene”[Title/Abstract] OR “Dental care”[Title/Abstract] OR “Oral health promotion”[Title/Abstract] OR “Oral hygiene instruction”[Title/Abstract]) AND (“intervention ^∗^”[Title/Abstract] OR “Health promotion”[Title/Abstract] OR “Educational program”[Title/Abstract] OR “Randomized controlled trial”[All Fields] OR “clinical trial”[All Fields] OR “random allocation”[Title/Abstract])
Scopus	TITLE‐ABS‐KEY (“disabilit ^∗^” OR “impair ^∗^” OR “disabled” OR “special needs” OR “functional limitation ^∗^") AND TITLE‐ABS‐KEY (“oral health” OR “dental health” OR “oral hygiene” OR “dental care” OR “oral health promotion” OR “oral hygiene instruction") AND TITLE‐ABS‐KEY (“intervention ^∗^” OR “health promotion” OR “educational program") AND TITLE‐ABS‐KEY (“randomized controlled trial” OR “clinical trial” OR “random allocation")
EBSCOhost	((TI “disabilit ^∗^” OR AB “disabilit ^∗^” OR TI “impair ^∗^” OR AB “impair ^∗^” OR TI “disabled” OR AB “disabled” OR TI “special needs” OR AB “special needs” OR TI “functional limitation ^∗^” OR AB “functional limitation ^∗^") AND (TI “oral health” OR AB “oral health” OR TI “dental health” OR AB “dental health” OR TI “oral hygiene” OR AB “oral hygiene” OR TI “dental care” OR AB “dental care” OR TI “oral health promotion” OR AB “oral health promotion” OR TI “oral hygiene instruction” OR AB “oral hygiene instruction") AND (TI “intervention ^∗^” OR AB “intervention ^∗^” OR TI “health promotion” OR AB “health promotion” OR TI “educational program” OR AB “educational program") AND (TI “randomized controlled trial” OR AB “randomized controlled trial” OR TI “clinical trial” OR AB “clinical trial” OR TI “random allocation” OR AB “random allocation"))
ProQuest	((ti(disabilit ^∗^ OR impair ^∗^ OR disabled OR “special needs” OR “functional limitation ^∗^") OR ab(disabilit ^∗^ OR impair ^∗^ OR disabled OR “special needs” OR “functional limitation ^∗^")) AND (ti(“oral health” OR “dental health” OR “oral hygiene” OR “dental care” OR “oral health promotion” OR “oral hygiene instruction") OR ab(“oral health” OR “dental health” OR “oral hygiene” OR “dental care” OR “oral health promotion” OR “oral hygiene instruction")) AND (ti(intervention ^∗^ OR “health promotion” OR “educational program") OR ab(intervention ^∗^ OR “health promotion” OR “educational program")) AND (ti(“randomized controlled trial” OR “clinical trial” OR “random allocation") OR ab(“randomized controlled trial” OR “clinical trial” OR “random allocation")))
Cochrane Library	(“oral health intervention” OR “oral hygiene intervention” OR “oral self‐care” OR “oral hygiene practices” OR “toothbrushing” OR “dental plaque control” OR “gingival health") AND (“adults with disabilities” OR “persons with disabilities” OR “special care needs” OR “disabled individuals” OR “independent adults with disabilities") AND (“randomized controlled trial” OR “RCT” OR “controlled clinical trial"): Title Abstract Keyword
OpenGray	(“disabilit ^∗^” OR “impair ^∗^” OR “disabled” OR “special needs” OR “functional limitation ^∗^") AND (“oral health” OR “dental health” OR “oral hygiene” OR “dental care” OR “oral health promotion” OR “oral hygiene instruction") AND (“intervention ^∗^” OR “health promotion” OR “educational program") AND (“randomized controlled trial” OR “clinical trial” OR “random allocation")

### 2.6. Selection Process

All retrieved records were imported into EndNote 20 for deduplication and then transferred to Excel for screening and data management. Two independent reviewers (NS and PH) first screened a 10% pilot sample of citations, yielding a Cohen’s kappa of 0.86, indicating strong agreement. They then independently screened all titles and abstracts, followed by full‐text articles for potentially eligible studies. No disagreements occurred regarding study inclusion; any minor discrepancies in data interpretation were resolved through discussion and author consensus. The screening process was completed in September 2024.

### 2.7. Data Collection Process

Data extraction was performed independently by two reviewers (NS and PH) in October 2024 using a standardized extraction form in Excel. Extracted information included publication details, study design and setting, participant characteristics (including disability type), intervention and comparator descriptions, outcome measures, follow‐up duration, and main findings. All extracted data were compiled in structured summary tables.

### 2.8. Data Extraction

For each included study, we extracted bibliographic details (author, year, and country), study design and setting, sample size and participant characteristics (including type of disability), and descriptions of the intervention and control conditions. Outcome data included clinical indices (e.g., plaque and gingival measures), behavioral outcomes (e.g., toothbrushing frequency and adherence to oral hygiene), and knowledge or attitudinal measures, as reported by the original authors. Where available, we also recorded follow‐up duration, effect estimates, percentage change, and statistical significance (*p*‐values). These data were organized into structured summary tables to facilitate comparison across studies and disability groups.

### 2.9. Quality Assessment and Certainty of Evidence

Two examiners (NS and PH) independently assessed the methodological quality and risk of bias using the Cochrane Risk of Bias tool for randomized trials (RoB 2.0) [31]. Disagreements were resolved through consensus with all authors. Risk‐of‐bias judgments were visualized using the ROBVIS application [32] as domain‐level and overall summary plots.

Certainty of evidence for each outcome was appraised qualitatively, taking into account RoB 2.0 ratings and the methodological rigor of the included studies. Studies judged at lower overall risk of bias were considered to provide more robust evidence, whereas those with high risk or multiple domains with “some concerns” contributed lower‐certainty evidence. These quality assessments informed the narrative synthesis in the Results and Discussion.

### 2.10. Synthesis Approach

Because of substantial clinical and methodological heterogeneity in disability types, intervention content, outcome measures, and follow‐up periods, statistical pooling was not feasible and meta‐analysis was not undertaken [33]. Instead, we conducted a structured narrative synthesis. First, included trials were grouped by intervention type (educational, behavioral, pharmacological, or combined) and by disability category (e.g., intellectual/developmental, psychiatric, physical/sensory, and neurological). Within these groups, we compared direction and magnitude of change in clinical indices, behavioral outcomes, and knowledge or attitudinal measures, taking into account follow‐up duration and overall risk‐of‐bias judgments. Patterns of effectiveness and inconsistency across studies were then summarized in the text and in Table 2, with greater weight given to trials judged at lower overall risk of bias [32, 33, 44].

**Table 2:** Characteristics, methodologies, and outcomes of included studies.

Study (country)	Population and setting	Intervention vs. control	Follow‐up	Outcome measures	Key findings/effect sizes	Attrition/adherence
Baram et al. 2020 (Denmark) [34]	Parkinson’s disease; *N* = 29; age 32 – 72; outpatient	Home‐based jaw exercises (JawTrainer) + lip/cheek training + oral hygiene instruction (OHI) vs. standard OHI	4 months	Jaw opening capacity; chewing time; debris index (DI)	Jaw opening + 8% (*p* = 0.002); chewing time −29% (*p* = 0.009); DI −67% (*p* = 0.001)	High adherence (69% – 90%)
Agarwal et al. 2019 (India) [35]	Schizophrenia; *N* = 111; adults; outpatient	Oral health education (OHE) with monthly calendar‐based reinforcement vs. standard care	4 weeks to 6 months	Oral hygiene index (OHI); knowledge–attitude–practice (KAP)	Brushing twice daily 23.2% vs. 5.4% (*p* < 0.05); OHI 2.28 vs. 2.98 (*p* < 0.02)	Not reported
Coutinho et al. 2023 (India) [36]	Visual impairment; *N* = 48 (young adults); community	Audio‐tactile performance (ATP) training + Braille vs. Braille alone	6 months	Plaque index (PI); Gingival Index (GI); oral health knowledge	PI and GI decreased more in intervention group (*p* < 0.05); knowledge gain greater in intervention group	Not reported
Kuo et al. 2020 (Taiwan) [37]	Severe mental illness; *N* = 68 enrolled; age 20 – 59; inpatient	12‐week (biweekly) group education + individualized behavioral modification vs. usual care	12 weeks	Plaque index (PI); oral health knowledge	PI 42.6 vs. 61.8 (*p* < 0.001); improved self‐care skills	14.7% attrition (58/68 completed)
Almomani et al. 2009 (USA) [38]	Severe mental illness; *N* = 50 enrolled; community	Motivational interviewing (MI) + oral health education (OHE) vs. OHE alone	8 weeks	Plaque index (PI); self‐regulation	PI decreased from 3.6 to 1.9 (*p* < 0.01); MI group improved more than control	16% attrition (42/50 completed)
Pannuti et al. 2003 (Brazil) [39]	Intellectual disability (“mental handicap”); *N* = 43; institutionalized adults; inpatient	0.5% chlorhexidine gel twice daily vs. placebo gel	8 weeks	Interdental Bleeding Index (IBI); plaque index (PI)	IBI 33.2% vs. 60.6% (*p* < 0.001); PI no significant difference	0% attrition (all completed)
Mun et al. 2014 (South Korea) [40]	Mental disorders; randomized *N* = 88; analyzed *N* = 73 (A 23 B 22 C 28); inpatient/outpatient	Flash‐based video + brochures (± professional brushing/toothpick method per group) vs. brochures alone	12 weeks	Patient Hygiene Performance index (PHP) to assess plaque index; xerostomia (salivary flow)	Plaque index decreased in all groups (*p* < 0.0001); video groups more effective than brochure alone (*p* = 0.036)	17% attrition (15/88); final *N* = 73
Ab Malik et al. 2018 (Malaysia) [41]	Stroke survivors; Trial 2 *N* = 86; inpatient	Powered toothbrush + 1% chlorhexidine gel vs. manual toothbrush + toothpaste	6 months	Oral yeast prevalence; oral pathogen counts	Yeast prevalence reduced (*p* < 0.05); *S. aureus* and gram‐negative bacilli reduced	≈37% attrition
Lam et al. 2013 (Hong Kong) [42]	Stroke patients; *N* = 102 enrolled; postdischarge	OHI + chlorhexidine mouthrinse (± assisted brushing) vs. OHI alone	Up to 6 months	Plaque index; gingival bleeding	Greater reductions in plaque and gingival bleeding in chlorhexidine groups (*p* < 0.001)	≈20% attrition (81/102 completed)
García‐Carrillo et al. 2016 (Spain) [43]	Intellectual disability; *N* = 64 in six clusters; mean age 34.5; community	Sonic powered toothbrush vs. manual toothbrush	6 months	Plaque index (PI); Gingival Index (GI)	Significant improvements over time (*p* < 0.001) with no between‐group differences for PI or GI	Not reported

## 3. Results

### 3.1. Study Selection

**Figure 1:**
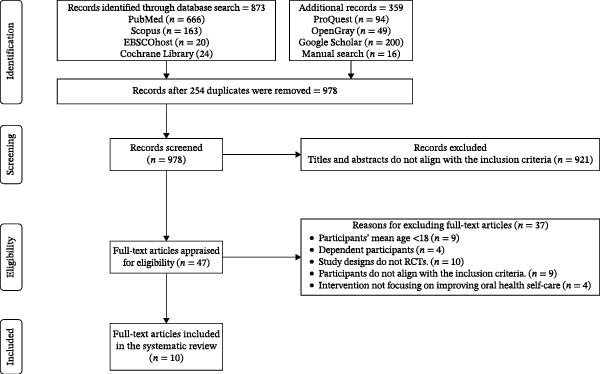
PRISMA 2020 flow diagram.

A total of 1232 records were identified through comprehensive database and manual searches (873 from databases, including PubMed, Scopus, EBSCOhost, and the Cochrane Library, and an additional 359 from other sources, such as ProQuest, OpenGrey, Google Scholar, and manual searches). After removing 254 duplicates, 978 titles and abstracts were screened. Of these, 921 were excluded for not meeting the eligibility criteria. The full texts of 47 articles were assessed, with 37 excluded for reasons such as ineligible study design (e.g., quasiexperimental or pilot studies), participant age below 18, dependence in daily activities, or interventions not focused on oral health self‐care. Ultimately, 10 RCTs, comprising 681 participants, met all inclusion criteria and were included in this review. The study selection process is detailed in the PRISMA 2020 flow diagram (Figure 1).

### 3.2. Characteristics of Included Studies

The 10 included RCTs were conducted in a variety of settings: Denmark [34], India [35, 36], Taiwan [37], the United States [38], Brazil [39], South Korea [40], Malaysia [41], Hong Kong [42], and Spain [43]. Study populations encompassed individuals with psychiatric disorders (five studies) [35, 37–40], neurological conditions (three studies) [34, 41, 42], intellectual disabilities (one study) [43], and physical impairments (one study) [36]. Sample sizes ranged from 29 [34] to 111 [35] participants. Participant ages spanned from early adulthood (with mean ages beginning at 20 years) to older adults (up to a mean of 69.9 years [42]). Gender distribution varied across studies, with most studies including a higher proportion of male participants, although two studies had more females [38, 40]. Study settings included community‐based care [36, 38, 43], inpatient facilities [37, 39, 41, 42], and outpatient clinics [34, 35], and one study involved both inpatient and outpatient participants [40]. A summary of each study, including population, intervention details, outcomes, and key findings, is presented in Table 2.

### 3.3. Risk of Bias Assessment and Certainty of Evidence

**Figure 2:**
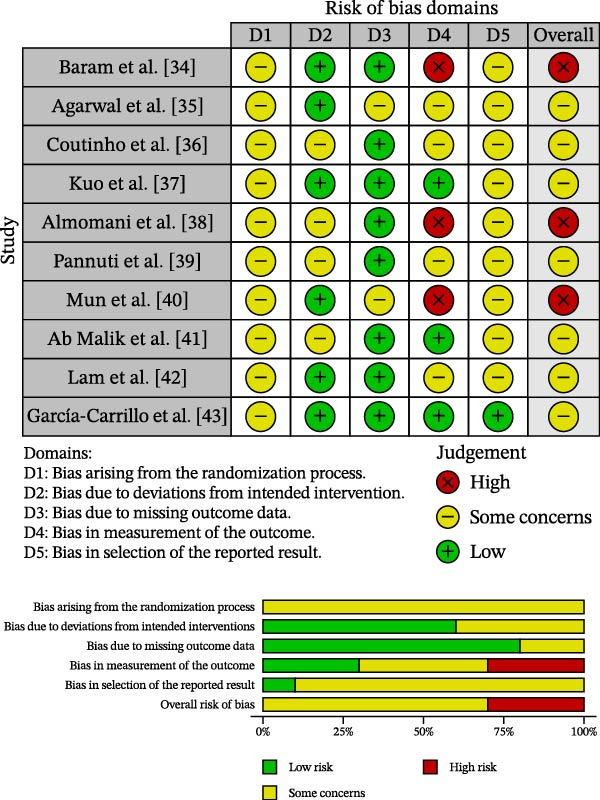
Risk of bias of RCTs included in the review.

As shown in Figure 2, assessment with the Cochrane RoB 2.0 tool indicated that none of the included studies met criteria for an overall “low risk” of bias. Seven RCTs were judged to have “some concerns” [35–37, 39, 41–43], while three were rated as having a “high risk” of bias overall [34, 38, 40], primarily due to issues in outcome measurement (lack of blinding) and selection of reported results. Therefore, the overall certainty of the findings is reduced. Certainty of evidence was judged qualitatively based on the RoB 2.0 ratings and the methodological quality of the included studies. Despite efforts to reduce publication bias through searches of multiple databases, gray literature, and manual sources, limiting inclusion to English‐language studies may have resulted in language bias [45].

### 3.4. Heterogeneity and Rationale for Narrative Synthesis

Meta‐analysis (including subgroup analyses) was not undertaken because the trials were clinically and methodologically heterogeneous. Clinically, studies enrolled heterogeneous disability groups (psychiatric, neurological, intellectual disability, and visual impairment) and were conducted across inpatient, outpatient, and community settings. Methodologically, intervention intensity and duration varied (4 weeks to 6 months), and outcomes were assessed using different indices and instruments (e.g., PI, GI, DI, IBI, xerostomia measures, and knowledge tools), with inconsistent reporting formats (means, percentages, and *p*‐values), limiting comparability and preventing statistical pooling [46]. We therefore used narrative synthesis to summarize patterns of effect and to explore potential reasons for differences across studies.

A formal, outcome‐level GRADE evidence profile was also not feasible because of substantial heterogeneity and incomplete reporting across outcomes. However, when interpreting findings, we considered key certainty domains (risk of bias, imprecision due to small samples, indirectness across populations/settings, and inconsistency in measurement and reporting), which suggest that confidence in effect estimates is generally limited for most outcomes [47].

### 3.5. Synthesis of Results

Studies were categorized by intervention type. Detailed characteristics and quantitative outcomes are presented in Table 2.

#### 3.5.1. Educational and Behavioral Interventions (Seven RCTs)

Across seven RCTs, educational and behavioral interventions generally improved oral hygiene and/or self‐care outcomes, although measures and follow‐up varied across trials [34–38, 40, 43]. In Parkinson’s disease, an education‐based program over 4 months increased jaw opening (8%; *p* = 0.002), reduced chewing time (29%; *p* = 0.009), and decreased the debris index (DI) (67%; *p* = 0.001) [34]. In adults with schizophrenia, OHE with calendar‐based reinforcement improved twice‐daily toothbrushing (23.2% vs. 5.4%; *p*  < 0.05) and oral hygiene index scores (2.28 vs. 2.98; *p*  < 0.02) versus standard care [35]. In young adults with visual impairment, audio‐tactile performance (ATP) plus Braille produced greater reductions in plaque and gingival indices and larger knowledge gains than Braille alone at 6 months (*p*  < 0.05) [36]. In inpatient severe mental illness settings, a 12‐week group education plus individualized behavioral modification program reduced plaque index (PI) compared with usual care (42.6 vs. 61.8; *p*  < 0.001) and improved self‐care behaviors [37]. Motivational interviewing (MI) added to OHE reduced PI (3.6 to 1.9; *p*  < 0.01) and was more effective than education alone [38]. A flash‐based video intervention combined with brochures and the toothpick method achieved greater plaque reduction than brochures alone at 12 weeks (*p* = 0.036) [40]. Where reported, confidence intervals were extracted; for example, in adults with intellectual disability, PI improved from baseline to 3 months (mean difference 0.453 and 95% CI 0.35 – 0.56) and to 6 months (mean difference 0.442 and 95% CI 0.33 – 0.55; both *p*  < 0.001), with no significant difference between powered and manual toothbrushes [43].

#### 3.5.2. Educational Plus Pharmacological Adjuncts (Two RCTs)

Two RCTs in stroke populations evaluated pharmacological adjuncts alongside oral hygiene instruction (OHI) [41, 42]. In a 6‐month inpatient trial, a powered toothbrush plus 1% chlorhexidine gel significantly reduced oral yeast prevalence and pathogenic bacterial counts compared with manual brushing and toothpaste (*p*  < 0.05) [41]. In a postdischarge trial with follow‐up up to 6 months, adding chlorhexidine mouthrinse (with or without assisted brushing) produced greater reductions in plaque and gingival bleeding than instruction alone (*p*  < 0.001) [42].

#### 3.5.3. Pharmacological‐Only Intervention (One RCT)

One 8‐week inpatient RCT in institutionalized adults with intellectual disability found that 0.5% chlorhexidine gel used twice daily significantly reduced interdental bleeding versus placebo (33.2% vs. 60.6%; *p*  < 0.001) but showed no significant between‐group difference in PI [39].

### 3.6. Attrition, Adherence, and Limitations of Long‐Term Evidence

Attrition and adherence were inconsistently reported across trials (Table 2). Where available, attrition ranged from 0% to ~37%, and one trial reported high adherence (69% – 90%). Because follow‐up in the included RCTs ranged from 8 weeks to 6 months, the durability of observed improvements beyond the short term remains uncertain [34–43], consistent with broader oral health intervention literature reporting short follow‐up periods and substantial heterogeneity in study design and outcome measures [48].

## 4. Discussion

This systematic review assessed the effectiveness of oral health interventions in adults with disabilities who are capable of performing their own oral health self‐care. Overall, the evidence indicates that multifaceted interventions, particularly those integrating structured education, behavioral reinforcement, and pharmacological adjuncts, are most beneficial for improving oral hygiene outcomes in this population. Nevertheless, sustaining long‐term adherence and ensuring lasting benefits remain challenging.

Educational and behavioral interventions were the most widely implemented and consistently effective strategies across diverse populations, including those with psychiatric disorders, neurological conditions, intellectual disabilities, and physical impairments [34–38, 40, 43]. These interventions incorporated structured OHE delivered through various accessible formats (e.g., audio‐tactile aids and Braille), demonstration‐based instruction, hands‐on training, MI, calendar‐based reinforcement, and personalized counseling. Consistently, these strategies enhanced oral health knowledge, attitudes, daily self‐care practices, and clinical outcomes such as plaque and debris indices. Regarding intervention characteristics, successful programs typically utilized durations ranging from 12 weeks to 6 months, allowing sufficient time for habit formation. Delivery modes that utilized multisensory approaches, such as ATP techniques and flash‐based videos, were particularly effective in overcoming sensory or cognitive barriers. Furthermore, the frequency of reinforcement was a critical factor; strategies employing regular monitoring, such as monthly calendar‐based checks or biweekly group sessions, showed stronger adherence and outcomes compared to single‐instruction methods. Our findings align with those of Menegaz et al. [49], who reported that educational interventions in health services, including oral health, foster positive behavioral change. The need for individualized reinforcement and ongoing behavioral support was particularly evident in people with psychiatric disorders, consistent with Lecomte et al. [50].

For adults with neurological conditions such as stroke and Parkinson’s disease, structured oral hygiene training and individualized instruction improved dexterity, oral function, and self‐care, with direct benefits for oral health and quality of life. This finding is supported by Auerbacher et al. [51], who advocate for early, individualized interventions in these populations. Individuals with intellectual disabilities benefited most from visual learning tools, written instructions, and hands‐on supervised brushing, supporting the conclusions of Waldron et al. [52] and Yeh et al. [53]. For those with physical disabilities, especially visual impairment, multimodal instructional approaches, including tactile, and Braille‐based education, proved most effective, consistent with Bhadauria et al. [24].

Behavioral interventions such as supervised brushing, token reinforcement, MI, and personalized counseling played a significant role in enhancing daily oral hygiene routines, particularly for those with psychiatric or intellectual disabilities, corroborating the benefits described by Vilar Doceda et al. [54] and Rojo et al. [55]. Practical implementation and sustainability were heavily influenced by the adaptability of the intervention to the participant’s daily routine and the level of caregiver involvement. For instance, high adherence rates (69% – 90%) were achieved when exercises were specifically tailored to the physical limitations of patients, such as those with Parkinson’s disease. Conversely, attrition rates reaching nearly 37% in longer‐term studies suggest that maintaining engagement without continuous external motivation or simplified protocols remains a significant challenge for sustainable implementation.

Educational and pharmacological interventions, particularly those combining structured education or behavioral support with chlorhexidine adjuncts, provided additional clinical benefits, especially for individuals with neurological impairments [41, 42]. These results align with current recommendations advocating multimodal, tailored strategies for complex populations [24, 56–59]. However, pharmacological agents should supplement, not replace, educational and behavioral strategies for the most robust and sustained outcomes.

Purely pharmacological interventions, such as chlorhexidine‐based regimens, demonstrated effectiveness in reducing plaque, gingival bleeding, and microbial load, particularly among adults with neurological or mental disabilities [39]. Chlorhexidine remains the gold standard for chemical plaque control [56–58], and its feasibility and utility in stroke populations are well supported [59]. However, the evidence underscores that these agents are most valuable when integrated into comprehensive educational and behavioral programs rather than used as stand‐alone solutions.

Given substantial clinical and methodological heterogeneity and inconsistent outcome reporting across trials, quantitative pooling was not appropriate, and the findings were therefore synthesized narratively. For the same reason, a formal outcome‐level GRADE assessment could not be applied; instead, confidence in the evidence was interpreted qualitatively with reference to risk‐of‐bias domains and key GRADE considerations [47].

## 5. Study Limitations

Several limitations should be noted. Considerable heterogeneity in study designs, sample sizes, intervention protocols, and target populations precluded meta‐analysis; therefore, a narrative synthesis was conducted. Although dual independent screening and data extraction were employed to reduce review bias, the included studies exhibited moderate to high risk of bias, particularly in outcome measurement and selective reporting domains. No included study was rated at low risk of bias overall. As a result, the certainty of evidence was moderate to low, and findings should be interpreted with caution. Other limitations include restriction to English‐language publications, which may have introduced language bias, and the potential for publication bias despite comprehensive database and gray literature searches. Additionally, while this review rigorously assessed risk of bias, a formal GRADE assessment was not feasible due to the inability to perform a meta‐analysis given the heterogeneity of data. Instead, the certainty of evidence was evaluated qualitatively based on the methodological rigor and risk‐of‐bias domains of the included trials. Finally, it is important to note that none of the included studies provided data on cost‐effectiveness or specific resource requirements. While educational materials and brief training may be low‐cost, programs involving supervised brushing, staff‐delivered behavioral modification, or MI require workforce time and training; assistive devices (e.g., powered toothbrushes) may increase upfront costs. Including economic and implementation evaluations alongside effectiveness outcomes would support policymakers and planners in judging scalability across inpatient, outpatient, and community settings.

## 6. Conclusion

This systematic review highlights the importance of tailored, multifaceted interventions to address the oral health needs of individuals with disabilities. Structured education, ongoing assessment, and long‐term support are vital for improving oral hygiene outcomes. Educational programs, especially when combined with behavioral reinforcement, can substantially enhance self‐care adherence, but sustained improvement requires continued engagement from caregivers and healthcare providers. Pharmacological interventions provide valuable adjunctive benefits but must be integrated into broader educational and behavioral frameworks. Interdisciplinary collaboration among healthcare providers, caregivers, and policymakers is essential for developing and sustaining comprehensive oral health interventions for this vulnerable population. Given the moderate to low certainty of the evidence, high‐quality, standardized trials with longer‐term follow‐up are needed to better guide clinical practice and policy.

## Author Contributions

**Nithimar Sermsuti-Anuwat:** conceptualization, methodology, data curation, formal analysis, investigation, funding acquisition, project administration, writing – original draft preparation, writing – review and editing. **Palinee Hongpaitoon:** data curation, formal analysis, validation, investigation, writing – review and editing. **Daophon Ardiam:** investigation, data curation, resources, writing – review and editing.

## Funding

This study was supported by grants for the development of new faculty staff, Ratchadaphiseksomphot Fund (Grant DNS 64_055_32_004_1), Chulalongkorn University, Bangkok, Thailand.

## Disclosure

All authors approved the final manuscript.

## Ethics Statement

Ethical approval was not required for this study as it is a systematic review of previously published literature and does not involve direct interaction with human or animal subjects.

## Conflicts of Interest

The authors declare no conflicts of interest.

## Data Availability

The data that support the findings of this study are available from the corresponding author upon reasonable request.
